# Age and regional differences in clinical presentation and risk of hospitalization for dengue in Brazil, 2000-2014

**DOI:** 10.6061/clinics/2016(08)08

**Published:** 2016-08

**Authors:** Marcelo N. Burattini, Luis F. Lopez, Francisco A.B. Coutinho, João B. Siqueira, Sheila Homsani, Elsa Sarti, Eduardo Massad

**Affiliations:** I Faculdade de Medicina da Universidade de São Paulo, Divisão de Informática Médica, São Paulo/SP, Brazil; II Hospital São Paulo, Escola Paulista de Medicina, São Paulo/SP, Brazil; III CIARA, Florida International University, Miami, Florida, United States of America; IV Universidade Federal de Goiás, Instituto Tropical de Patologia e Saúde Pública, Goiânia/GO, Brazil; V Sanofi Pasteur, São Paulo/SP, Brazil; VI Sanofi Pasteur Latinoamerica, Mexico City, Mexico; VII London School of Hygiene and Tropical Medicine, London, UK

**Keywords:** Dengue, Morbidity, Epidemiology, Surveillance System, Hospitalization, Brazil

## Abstract

**OBJECTIVES:**

Dengue cases range from asymptomatic to severe, eventually leading to hospitalization and death. Timely and appropriate management is critical to reduce morbidity. Since 1980, dengue has spread throughout Brazil, affecting an increasing number of individuals. This paper describes age and regional differences in dengue’s clinical presentation and associated risk of hospitalization based on more than 5 million cases reported to the Brazilian Ministry of Health from 2000-2014.

**METHODS:**

We performed a retrospective analysis of ∼5,450,000 dengue cases, relating clinical manifestations and the risk of hospitalization to age, gender, previous infection by dengue, dengue virus serotype, years of formal education, delay to first attendance and the occurrence of dengue during outbreaks and in different Brazilian regions.

**RESULTS:**

Complicated forms of dengue occurred more frequently among those younger than 10 years (3.12% *vs* 1.92%) and those with dengue virus 2 infection (7.65% *vs* 2.42%), with a delay to first attendance >2 days (3.18% *vs* 0.82%) and with ≤4 years of formal education (2.02% *vs* 1.46%). The risk of hospitalization was higher among those aged 6-10 years old (OR 4.57; 95% CI 1.43-29.96) and those who were infected by dengue virus 2 (OR 6.36; 95% CI 2.52-16.06), who lived in the Northeast region (OR 1.38; 95% CI 1.11-2.10) and who delayed first attendance by >5 days (composite OR 3.15; 95% CI 1.33-8.9).

**CONCLUSIONS:**

In Brazil, the occurrence of severe dengue and related hospitalization is associated with being younger than 10 years old, being infected by dengue virus 2 or 3, living in the Northeast region (the poorest and the second most populated) and delaying first attendance for more than 2 days.

## INTRODUCTION

Over the past 50 years, dengue has become one of the top priorities of the World Health Organization (WHO) in terms of global health. This infection occurs in more than 100 countries and is estimated to affect 50-100 million people, with 500,000 severe cases demanding hospitalization and approximately 20,000 deaths every year 1. More than 200 disability-adjusted life years per million population are lost per year 2, with an estimated cost ranging from US$248-571 for ambulatory and hospitalized cases in certain Asian and Latin American countries, implying an annual cost as high as US$500-1000 million per year in countries such as Brazil 3.

Since its reappearance in Brazil in the early 1980s, dengue has progressively spread throughout the country, affecting an increasing number of individuals, states and municipalities 3-7. Initially, it presented as recurrent yearly epidemics with a relatively small number of cases that affected different regions of the country and that occasionally spared the same region one year but not another. However, the increasing circulation of various serotypes of the virus has been associated with increases in the frequency and extent of epidemics, with increasing numbers of complicated dengue cases and hospitalizations 3-6.

Human infection by dengue virus (DENV) leads to a wide spectrum of clinical presentations, often with unpredictable clinical evolution and outcomes 8. Classic dengue fever (DF) is an acute febrile illness, with a sudden onset of fever typically after 5-7 days (full range from 3-12 days) of incubation. The accompanying symptoms are uncharacteristic and the most frequently reported findings are headache and generalized muscular, articular and osseous pain. Occasionally, retro-orbital pain; photophobia; a mild maculopapular skin rash; and minor hemorrhagic manifestations, such as petechiae, ecchymosis, epistaxis and positive tourniquet testing, are also reported 8-10.

However, a small proportion (∼5%) of patients may present or evolve to more severe clinical forms, mostly characterized by plasma leakage with or without hemorrhage, known as dengue hemorrhagic fever (DHF); dengue shock syndrome (DSS); or other complicated clinical forms that have significant morbidity and mortality 8-10. Warning signs of progression to severe dengue usually occur after the first few days of the febrile disease and include severe abdominal pain; persistent vomiting; difficulty breathing; signs of hypovolemic shock; a rapid decline in the platelet count and an increase in hematocrit of at least 10%, with or without mucosal bleeding 8-10.

Early clinical findings are nonspecific and demand a high index of suspicion because recognizing the early signs of potential complications or shock and promptly initiating intensive supportive therapy can reduce the risk of death among patients with severe dengue from 10% to <1% 8,9. This is particularly important during dengue outbreaks, when health services need to cope with the sudden surge in demand 8,9.

In this paper, we present a clinical-epidemiological description of dengue in Brazil based on a reanalysis of nearly 5,450,000 dengue cases reported to the National Epidemiological Surveillance Secretary (SVS) of the Brazilian Ministry of Health (MoH) and assessed by the Brazilian national reportable disease information system, named SINAN (Sistema de Informação de Agravos de Notificação) 11, from 2000-2014. This analysis focuses specifically on age, gender, DENV serotype, the time to first attendance, the number of years of formal education and regional differences in the clinical presentation of dengue and risk factors related to hospitalization.

In 1997, the WHO proposed the grouping of symptomatic DENV infections into three categories: undifferentiated fever, DF and DHF. DHF was further classified into four severity grades, with grades III and IV being defined as DSS 8. However, many have complained that this classification is difficult to use in different clinical settings 12-14, as summarized in a systematic literature review 15. More recently, the WHO conducted a study aimed to *“...devise a system that identified patients requiring major intervention with sufficient sensitivity and specificity to be practically useful"* 16. However, as mentioned by the authors, only a small fraction of cases evolved to severe disease and *“...much larger studies are necessary to fully characterize features associated with disease progression"* 16. This issue notwithstanding, the results of the study were incorporated into the 2009-10 WHO simplified classification for dengue, which defined three categories: DF, dengue with warning signs and severe dengue 8,16.

However, the frequency of clinically complicated dengue cases increased, leading to high morbidity and increased hospitalization. The cases did not fulfill the strict WHO criteria for dengue with warning signs or severe dengue, leading the Brazilian MoH to propose a new category of severe dengue, named “dengue with complications” (or CD, an acronym for “complicated dengue”) 10. CD was characterized as all severe dengue cases that did not fulfill the WHO criteria for dengue with warning signs, DHF or DSS and those that presented with any one of the following signs and symptoms: severe alterations in the central nervous system (CNS), cardiorespiratory dysfunction, hepatic or renal insufficiency, a platelet count below 20,000/mm^3^, gastrointestinal bleeding, pleural effusion or ascites, a global white blood cell count below 1,000/mm^3^, or any dengue case that led to death without having been characterized as DHF or DSS 10,17. This classification was officially adopted in Brazil and therefore, the cases notified to SINAN are classified accordingly.

Previous studies have qualitatively described the occurrence of dengue and DHF in Brazil since the early 1980s 18, but none has provided a systematic analysis of the clinical-epidemiological-social aspects of dengue occurrence in Brazil.

**Figure 1:**
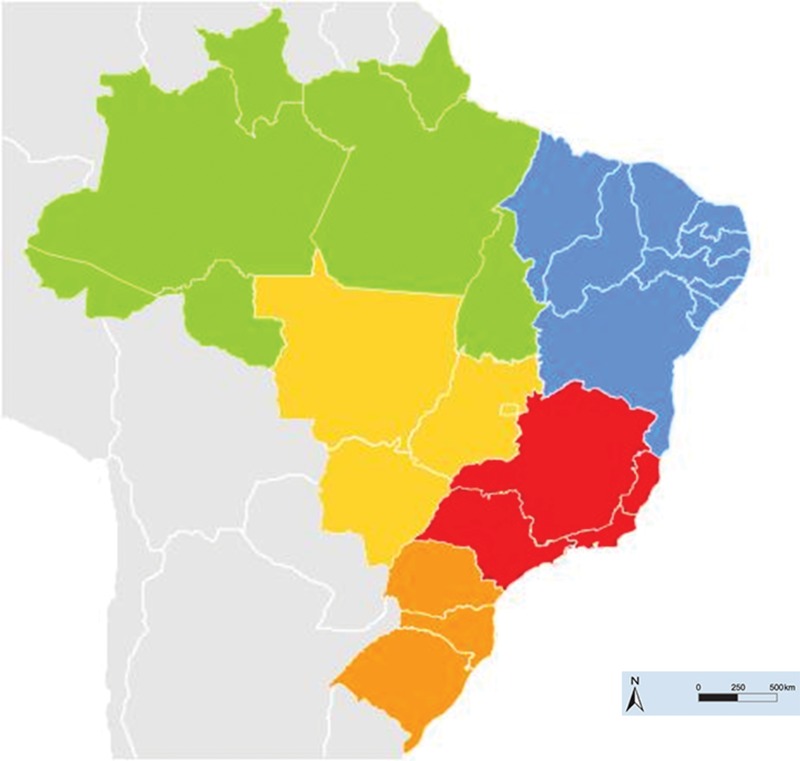
Geo-political map of Brazil, with colored representations of its geographic regions (green for North, blue for Northeast, red for Southeast, yellow for Central-West and orange for South).

Brazil is the fifth largest country in the world in terms of both territory and population size. It is divided into 26 states and the Distrito Federal, an independent territory seat for the federal capital, Brasília and certain surrounding municipalities. The 26 states are grouped into five distinct geographical regions that share geographical, physical, ethnic, cultural and economic similarities, known as the North region (with 7 states), the Northeast region (with 9 states), the Southeast region (with 4 states) and the Central-West and South regions (with 3 states each) (see Figure 1).

Brazil has drastically urbanized since the mid-1970s and now, more than 78% of the Brazilian population lives in an urban setting. The North region is nearly entirely in the Amazon and most of it is covered by tropical rain forest. The Northeast region is the poorest in the country and most of it is in a semi-arid area, with sparse rainfalls and poor socio-demographic conditions. Meanwhile, the Southeast region is the most populated and developed region in the country. The South region is characterized by a moderate climate (colder than the rest of the country) and therefore has been spared (with the exception of Paraná state) from dengue epidemics. Finally, the Central-West region is an area with a rich agricultural-based economy on the southern border of the Amazon. Table 1 summarizes several socio-demographic characteristics of Brazil and its regions 19 that are related to dengue transmission and to the risk of hospitalization due to dengue.

## METHODS

This was a population study based on a secondary data analysis of dengue morbidity and hospitalization risk. Unidentified data from dengue cases reported to the Health Surveillance Secretary of the Brazilian MoH, as assessed by the Brazilian national reportable disease information system, named SINAN (Sistema de Informação de Agravos de Notificação) 11 were specifically analyzed. Since its reintroduction in Brazil in the early 1980s, it is mandatory that all dengue cases are notified to SINAN 10,18.

According to the Brazilian MoH classification, DF is defined as an acute febrile illness no more than seven days long, accompanied by at least two of the following symptoms: headache, retro-orbital pain, myalgia, arthralgia, prostration and exanthema, possibly associated with minor hemorrhagic manifestations. In addition, the patient should have been in an area with active dengue transmission in the last 15 days 10.

All reported dengue cases that occurred in municipalities in which dengue epidemics occurred in the period from 2000-2014 were selected for this analysis. A dengue epidemic was defined as the period of time in which the following conditions were simultaneously satisfied:

1. Having had three consecutive weeks with an increasing number of cases;
2. The first week of the outbreak was the first in which the number of cases surpassed the 95% upper limit of the confidence interval of the average number of cases in the previous 5 weeks; and
3. The last week of the epidemic was the first in which the number of cases dropped below the previously mentioned limit after three consecutive weeks with a decreasing number of cases.

The resulting database comprised 5,444,285 dengue cases, corresponding to nearly 80% of the total dengue cases reported to the Brazilian MoH in the study period. The other ∼20% of cases occurred in municipalities without dengue epidemics in the period analyzed (as defined above).

The clinical manifestations of dengue at presentation were related to age, gender, previous infection by DENV, DENV serotype and Brazilian geographic region and were expressed as absolute counts and percentages of patients who presented with each of the symptoms and signs reported. The prevalence of dengue, DHF, DSS and CD, as defined by the Brazilian MoH, were also related to the same variables. Finally, the risk of hospitalization was related to the same variables and to years of formal education, delay to first attendance (in days), a previous dengue episode and whether the dengue case occurred during an outbreak/epidemic.

Associations between variables were assessed by means of chi-square and Bonferroni tests and a multivariate logistic regression analysis was performed to identify the main risk factors related to hospitalization. The analysis was conducted using Statistica 64 (V12)^®^, StatSoft, INC. and the significance level was set at 5%.

### Ethics Statement

This research involved the use of anonymized patient medical data extracted from the Brazilian reportable disease information system and was approved by the Ethics in Research Committee of the Federal University of São Paulo, Brazil (#2059131213).

## RESULTS

**Figure 2:**
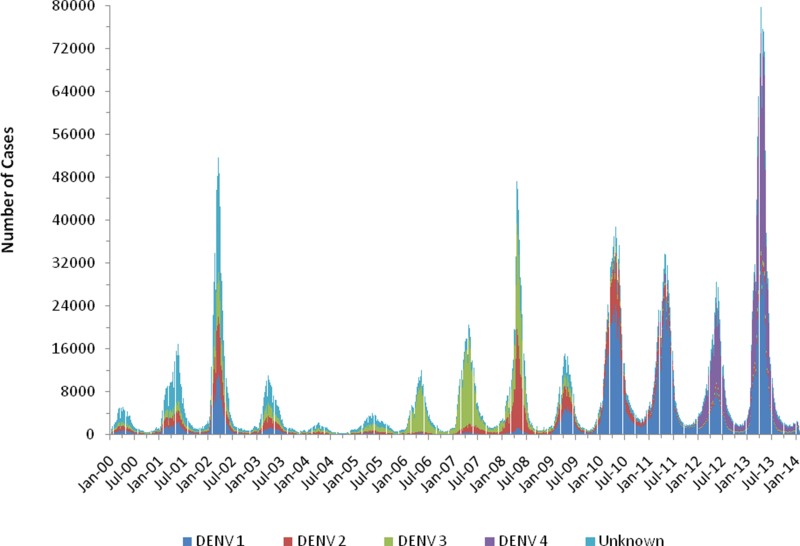
Dengue occurrence per epidemiological week and serotype in Brazil from 2000-2014.

**Figure 3:**
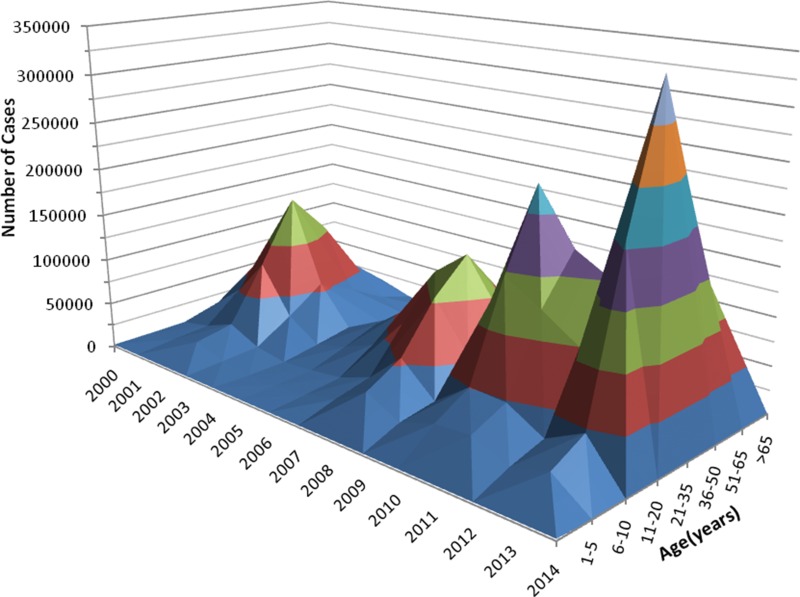
Successive dengue epidemics in Brazil from 2000-2014, as related to the affected age class and year of occurrence.

Figure 2 shows the occurrence of dengue as related to the main serotypes involved and the epidemiological week per year between 2000 and early 2014, and Figure 3 shows the successive dengue epidemics as related to the age classes affected during the period from 2000-2014.

Table 2 shows the frequency of dengue symptoms as related to the selected variables. Fever was experienced by approximately 95-97% of the patients. Headache was experienced by approximately 90% but showed some variation, with lower frequency among children below 10 years of age and adults above 65 years. Headaches were also less pronounced in males and in people from the North and Northeast regions. In contrast, diarrhea and rash were the least frequent symptoms and showed marked differences (21-45%) in their frequency among the distinct classes of variables. Rash was more frequent in children younger than 5 years old, those infected by DENV 1 and those living in the North region. Meanwhile, diarrhea was more frequent among adults older than 50 years and those living in the North region. The others symptoms varied in frequency from 38-88%, showing marked variations among the different groups (see Table 2 for details).

Table 3 shows the number of dengue cases and the frequency of its different clinical forms as related to the selected variables. It is noteworthy that the highest frequencies of CD, DHF and DSS were related to younger age classes, DENV types 2 and 3, the North and Northeast regions, a delay to first attendance greater than 3 days and less than 4 years of formal education.

Table 4 shows the univariate analysis of the risk of hospitalization as related to the selected variables. During the period analyzed, 113,726 patients with age information were hospitalized, with ∼60% of them aged from 11-50 years. This finding notwithstanding, the highest proportion of hospitalizations was observed among the youngest patients, with more than 15% of children younger than 10 years old being hospitalized. Additionally, a relatively high proportion of elders (>65 years old) were hospitalized (12.4%) due to dengue. Regarding the DENV serotypes, the highest proportion of hospitalizations (25.5%) was related to DENV 2, followed by DENV 3 (16.8%). The Northeast region was the region with the highest (13.5%) hospitalization rate in Brazil. Delaying the first attendance by more than two days was related to an increase in the risk of hospitalization (the risk nearly tripled for delays between 3-5 days and nearly quintupled for delays greater than 5 days). Having had less than four years of formal education increased the risk of hospitalization by 1.5 times. Finally, having had dengue during a period not defined as a dengue epidemic nearly doubled the risk of hospitalization.

Table 5 shows a summary of the multivariate analysis of the risk of hospitalization due to dengue. All variables described in Table 4 were included in the logistic model, as they were all significantly associated with the risk of hospitalization. It is noteworthy that those aged less than 5 years had nearly triple the risk and that those aged between 6-10 years had nearly quintuple the risk. Moreover, people older than 65 years had nearly double the risk of hospitalization, although this result was not significant in the multivariate analysis. With respect to dengue serotypes, DENV 2 was associated with a six times higher risk of hospitalization than for DENV 4, while DENV 3 was associated with a nearly two times higher risk than for DENV 4. In contrast, DENV 1 was associated with only one third of the risk of hospitalization for DENV 4 (this result was not significant in the multivariate analysis). With respect to the different Brazilian regions, the Northeast region was associated with a 38% higher risk of hospitalization relative to the South region. In contrast, both the Southeast and the Central-West regions had lower risks (88% and 41%, respectively) of hospitalization compared with the South region. Finally, delaying the first attendance by more than 7 days was associated with a nearly four times higher risk of hospitalization compared with a delay of less than 2 days. In addition, compared with a delay of less than 2 days, a delay between 5 and 7 days increased the risk by 2.5 times.

## DISCUSSION

Infection by any serotype of DENV results in a large spectrum of nonspecific clinical manifestations with an unpredictable clinical course and outcome, ranging from asymptomatic cases to severe clinical forms leading to hospitalization, a need for intensive care treatment and death. Timely and appropriate monitoring and clinical management of dengue patients, mainly entailing early fluid replacement interventions, is critical to reduce morbidity and mortality 8,10.

DENV and its vectors are now widely distributed throughout tropical and subtropical regions, spreading particularly over the last half-century and threatening even temperate regions, such as North America and Europe 20. Throughout the world, the significant geographic expansion of dengue has been coupled with rapid increases in the numbers of cases and epidemics, leading to an increasing number of more severe forms of dengue, hospitalizations and deaths 20. In the Southeast Asia (SEA) and Western Pacific (WP) regions, the expansion of dengue has occurred over the past few decades; epidemics are occurring persistently in regular 3- to 5-year cycles, with an increasing number of reported cases in many countries that are now classified as hyperendemic and all four DENV serotypes being reported as present 20. Severe dengue is endemic in most SEA countries and is a leading cause of hospitalization and death in children from the region, which has reported rates of severe dengue up to 18 times higher than in the Americas 20.

Also in the Americas, dengue transmission resurged in the late 1970s, and now many countries are hyperendemic, with epidemics occurring cyclically every 3-5 years, as in SEA; these epidemics are also increasing in frequency and size, particularly in Latin America 20. Similarly, since the early 1980s, dengue has spread throughout Brazil, occurring as annual recurrent seasonal epidemics mainly in the summer, with an increasing number of complicated cases and hospitalizations. All four serotypes of DENV circulate in Brazil, and there is a clear tendency towards an increase in the number of children and youth affected (see Figure 3), as previously observed in SEA. In 2013 and 2015 two of the largest epidemics of dengue ever recorded in a single country occurred in Brazil, with both involving more than 1,300,000 cases and with the second peaking at more than 1,600,000 cases notified to the Brazilian MoH 11.

This paper describes age and regional differences in the clinical presentation of dengue among more than 5.4 million cases reported to the SINAN in the period between 2000 and 2014. It also presents an analysis of the risk of hospitalization due to dengue. We found that the highest frequencies of CD, DHF and DSS were related to younger age classes, to DENV types 2 and 3, to the North and Northeast regions, to a delay to first attendance greater than 2 days and to less than 4 years of formal education. In addition, being younger than 10 years old nearly quintupled the risk of hospitalization. Infection by DENV 2 was associated with a risk of hospitalization six times larger than that for DENV 4, and DENV 3 was associated with a risk nearly twice as large as for DENV 4. Regarding the different Brazilian regions, the Northeast region was associated with a 38% increase in the risk of hospitalization relative to the South region. In contrast, both the Southeast and the Central-West regions had lower risks (88% and 41%, respectively) of hospitalization. Finally, delaying the first attendance by more than 5 days was associated with a nearly four times greater risk of hospitalization compared with a delay of less than 2 days.

Dengue usually presents as recurrent, seasonal, yearly epidemics, which may increase the awareness of the health sector, which may in turn promptly suspect and identify clinical cases 4. However, Brazil is a large country, with dengue occurring differently in different regions, states and municipalities 4-6. In certain areas, recurrent yearly dengue epidemics and outbreaks are common, occurring approximately during the same period year after year. In other regions, however, dengue presents as irregular large epidemics occurring sparsely over time and with a large number of cases occurring year round (but mainly throughout the summer and autumn), but without relation to recognizable outbreaks or epidemics. In these regions, the clinical awareness that prompts dengue suspicion and diagnosis may not be as present as needed. Whether dengue occurred during an outbreak/epidemic seems to be an important factor, with our study showing that the risk of hospitalization nearly doubles for dengue cases not related to recognizable outbreaks/epidemics. This finding indicates *per se* the need for adequate descriptions of age, gender and regional differences in the clinical presentation of dengue, as presented in this paper.

Timely and appropriate monitoring and clinical management of dengue cases depend on many parameters; among the most important are the socio-educational level of the population, the clinical awareness of physicians and other health personnel and the adequate infrastructure of the health system. Our results, sadly but not surprisingly, point to the Northeast region, which is the poorest and the second most populated in the country, as the region with the highest risk of hospitalization due to dengue. It is followed by the North region, which is nearly equally poor and destitute of basic conditions for adequate population care. Health conditions related to impoverishment and low socio-economic development, such as malnutrition and poor urban conditions and public services (including poor waste collection, water supply and sanitation), together with a poor basic public health structure constitute a nearly ideal amalgam for the breeding of *Aedes* spp. mosquitoes and the development of severe dengue cases as dengue outbreaks and epidemics subsequently occur.

Previous studies have described and critically commented on the clinical presentation of dengue in Brazil and elsewhere 12,14,18. Socio-demographic characteristics have also been previously studied in relation to the occurrence of dengue, but not in relation to its clinical presentation or the risk of hospitalization due to dengue 21-25. Therefore, to the best of our knowledge, this is the first study to systematically analyze and describe regional differences in dengue’s clinical presentation and associated risk of hospitalization and to relate these different presentations and outcomes to distinct socio-demographic variables.

Certain limitations of this study are related to its retrospective nature because information related to important data may be incomplete, which is a typical limitation of this type of study. In addition, several other important characteristics, such as nutritional status and individual socio-economic variables, that could be associated with the risk of hospitalization could not be assessed in the present analysis. Finally, dengue mortality and the risk factors related to the lethality of dengue were not analyzed here, as they will be specifically analyzed in a future publication.

## AUTHOR CONTRIBUTIONS

M.N. Burattini planned the study, analysed data, discussed results and write the manuscript; L.F. Lopez, F.A.B. Coutinho and E. Massad analysed data, discussed results and write the manuscript; J.B. Siqueira-Jr formatted the data base and contributed to data analysis, discussion of the results and writing of the manuscript; S. Homsani and E. Sarti contributed in the discussion of results and writing of the manuscript.

## Figures and Tables

**Table 1:** Socio-demographic characteristics of the population in Brazil and in the Brazilian regions 19.

Socio-Demographic Characteristic	Brazil	Brazilian Regions				
		North	Northeast	Southeast	South	Central-West
Estimated population in 2014	203,834,190	16,394,272	57,454,690	85,663,161	29,580,665	14,741,402
Urbanization rate (%)	84.8	74.6	73.3	93.2	85.5	90.1
≈ Number of dwellings (1000)	57,557	4,010	14,994	25,310	8,993	4,250
% without regular water supply	18.3	44.6	24.0	9.0	11.6	16.2
% without adequate sanitation	44.5	84.8	66.3	14.1	42.1	56.4
% without regular waste collection	14.1	25.8	26.7	4.4	5.8	8.6
% not covered by the family health program	30.1	49.0	35.2	64.1	49.7	50.9
Per capita GDP in US$ PPP (2014)	8,619	5,725	4,203	11,478	9,748	11,845
Gini index	0.526	0.526	0.530	0.511	0.481	0.544
Women’s fecundity rate	1.77	2.22	1.89	1.63	1.62	1.74
Infant mortality rate (per 1,000 born alive)	15.0	19.2	19.4	11.6	10.4	15.6
Life expectancy at birth (years)	74.8	71.5	72.2	76.6	76.9	74.4

**Table 2:** Frequency of dengue symptoms at presentation, as related to the selected variables.

	Fever	Headache	Rash	Generalized Pain	Prostration	Myalgia	Arthralgia	Nausea	Diarrhea
**Age (years)**									
1-5	95.4%	68.1%	49.6%	42.1%	60.1%	56.2%	37.5%	52.9%	26.7%
6-10	96.2%	87.5%	40.3%	59.5%	66.3%	69.6%	49.5%	59.5%	21.7%
11-20	96.2%	92.5%	36.0%	73.2%	70.5%	81.0%	62.8%	59.3%	22.9%
21-35	95.9%	93.4%	37.6%	77.1%	71.6%	86.0%	70.9%	61.2%	26.3%
36-50	96.0%	92.9%	41.8%	77.3%	74.2%	87.6%	74.2%	62.2%	29.2%
51-65	95.8%	91.5%	40.0%	73.9%	75.5%	87.6%	74.8%	63.0%	31.7%
>65	94.6%	88.1%	33.8%	67.8%	74.5%	85.7%	72.4%	60.0%	30.8%
**Gender**									
Female	93.5%	90.3%	40.1%	72.7%	69.3%	82.6%	67.5%	62.1%	26.3%
Male	94.3%	88.5%	34.0%	68.9%	67.3%	80.7%	64.2%	52.7%	25.7%
**DENV Serotype**									
DENV 1	97.4%	92.4%	45.1%	76.3%	76.4%	83.6%	73.8%	53.3%	22.7%
DENV 2	95.0%	92.6%	33.2%	76.3%	76.4%	85.0%	72.4%	57.3%	24.1%
DENV 3	98.2%	92.9%	33.0%	75.3%	74.5%	86.9%	72.5%	59.1%	23.1%
DENV 4	96.3%	92.2%	36.1%	73.4%	72.8%	83.3%	72.6%	64.4%	22.3%
**Brazilian Region**									
North	94.4%	89.6%	41.9%	71.4%	53.5%	81.6%	71.1%	52.9%	29.7%
Northeast	88.3%	83.4%	36.8%	61.6%	53.1%	73.4%	55.3%	47.3%	21.2%
Southeast	96.7%	93.0%	38.1%	76.8%	80.3%	86.4%	70.2%	65.2%	27.9%
Central-West	97.3%	92.1%	31.9%	74.0%	79.9%	86.0%	73.4%	64.1%	27.3%
South	94.7%	92.5%	31.5%	71.2%	77.5%	86.2%	67.1%	65.9%	24.5%
**Previous Dengue**									
Yes	97.6%	95.7%	36.7%	82.2%	77.5%	87.3%	75.6%	65.4%	29.2%
No	97.3%	93.0%	40.7%	76.5%	74.5%	85.3%	71.0%	61.8%	28.2%

**Table 3:** Frequency of the different clinical forms of dengue, as related to the selected variables.

	Dengue Cases	Classic Dengue	Complicated Dengue (CD)	Dengue Hemorrhagic Fever (DHF)	Dengue Shock Syndrome (DSS)
**Age (years)**					
1-5	158616	96.87%	2.29%	0.83%	0.02%
6-10	258372	94.75%	3.94%	1.28%	0.02%
11-20	801997	97.65%	1.85%	0.49%	0.01%
21-35	1270950	98.37%	1.32%	0.30%	0.01%
36-50	879684	98.02%	1.59%	0.38%	0.01%
51-65	462687	97.57%	2.00%	0.41%	0.02%
>65	163873	96.54%	2.86%	0.56%	0.04%
**Gender**					
Female	2408847	97.77%	1.76%	0.46%	0.01%
Male	1913157	97.47%	2.01%	0.50%	0.02%
**DENV Serotype**					
DENV 1	11546	95.96%	2.41%	1.51%	0.13%
DENV 2	2711	85.57%	7.65%	5.53%	1.25%
DENV 3	4416	91.04%	5.59%	3.15%	0.22%
DENV 4	3922	95.73%	2.45%	1.20%	0.61%
**Brazilian Region**					
North	446450	98.38%	1.03%	0.57%	0.02%
Northeast	999774	97.60%	1.65%	0.73%	0.02%
Southeast	2239451	97.54%	2.09%	0.35%	0.01%
Central-West	547436	97.25%	2.17%	0.57%	0.01%
South	90775	99.38%	0.36%	0.25%	0.01%
Previous Dengue					
Yes	105965	90.7%	1.3%	0.6%	0.0%
No	786398	93.1%	1.0%	0.4%	0.0%
**Delay to First Attendance (days)**					
0-2	1312749	99.01%	0.82%	0.16%	0.01%
3-5	964121	97.33%	2.10%	0.55%	0.02%
6-7	345171	95.21%	3.71%	1.05%	0.03%
>7	328240	95.86%	3.20%	0.91%	0.03%
**Years of Formal Education**					
≤4 years	336604	97.17%	2.02%	0.79%	0.01%
>4 years	1801282	98.06%	1.46%	0.46%	0.01%

**Table 4:** Results of the univariate analysis of the risk of hospitalization due to dengue, as related to the selected variables.

	# Hospitalized	% Hospitalized	*p*-value
**Age (years)**			
1-5	7485	13.87%	<0.0001
6-10	16573	17.47%	
11-20	23897	8.11%	
21-35	25142	5.64%	
36-50	19571	6.38%	
51-65	13276	7.61%	
>65	7782	12.37%	
**Gender**			
Female	61541	7.68%	<0.0001
Male	55389	8.50%	
**DENV Serotype**			
DENV 1	708	11.55%	<0.001
DENV 2	309	25.52%	
DENV 3	313	16.84%	
DENV 4	267	8.55%	
**Brazilian Region**			
North	10506	9.22%	<0.0001
Northeast	30000	13.47%	
Southeast	55275	4.91%	
Central-West	18707	6.32%	
South	2483	9.70%	
**Previous Dengue**			
Yes	50404	10.69%	<0.0001
No	369147	10.42%	
**Delay to First Attendance (days)**			
0-2	22761	3.80%	<0.0001
3-5	41214	9.06%	
6-7	25656	15.24%	
>7	21494	13.33%	
**Years of Formal Education**			
≤4 years	11712	11.41%	<0.0001
>4 years	31812	7.67%	
**During Outbreak/Epidemic**			
Yes	90576	6.96%	<0.0001
No	77101	12.21%	

**Table 5:** Summary of the multivariate analysis of the risk of hospitalization due to dengue, as related to the selected variables.

	Odds Ratio	Lower 95% CI	Upper 95% CI	*p*-value
Intercept 1	14.05	7.74	25.51	0.0001
**Age (years)**				
1-5	2.87	1.40	5.86	0.0385
6-10	4.57	1.43	29.96	0.0039
21-35	1.00	---	---	---
36-50	1.31	0.76	2.84	0.1035
>65	1.81	0.90	3.64	0.0958
**DENV Serotype**				
DENV 1	0.32	0.22	2.14	0.0747
DENV 2	6.36	2.52	16.06	0.0001
DENV 3	1.94	1.17	3.20	0.0100
DENV 4	1.00	---	---	---
**Brazilian Region**				
South	1.00	---	---	---
Southeast	0.12	0.08	0.18	0.0001
Central-West	0.59	0.43	0.82	0.0018
Northeast	1.38	1.11	2.10	0.0138
**Delay to First Attendance (days)**				
0-2	1.00	---	---	---
3-5	0.40	0.28	1.72	0.0871
6-7	2.49	1.33	4.66	0.0044
>7	3.62	1.62	8.09	0.0017

Obs: Only the variable categories that remained in the final model are shown.
